# Pro-Dopamine Regulator – (KB220) to Balance Brain Reward Circuitry in Reward Deficiency Syndrome (RDS)

**Published:** 2017-04-28

**Authors:** Kenneth Blum, Marcelo Febo, Lyle Fried, David Baron, Eric R. Braverman, Kristina Dushaj, Mona Li, Zsolt Demetrovics, Rajendra D. Badgaiyan

**Affiliations:** 1 Department of Psychiatry & McKnight Brain Institute, University of Florida College of Medicine, Gainesville, FL, USA; 2 Division of Addiction Services, Dominion Diagnostics, LLC, North Kingstown, RI, USA; 3 Igene LLC, Austin, TX, USA; 4 Departments of Psychiatry & Behavioral Sciences, Keck School of Medicine of USC, Los Angeles, CA, USA; 5 Division of Neuroscience Research & Addiction Therapy, Shores Treatment & Recovery Center, Port Saint Lucie, FL, USA; 6 Human Integrated Services Unit, University of Vermont Centre for Clinical & Translational Science, College of Medicine, Burlington, VT, USA; 7 Eötvös Loránd University, Institute of Psychology, Budapest, Hungary; 8 Division of Clinical Neurology, PATH Foundation NY, New York, NY, USA; 9 Division of Addiction Research & Therapy, Nupathways, Innsbrook, MO, USA; 10 Department of Psychiatry, Wright State University School of Medicine, Dayton, OH, USA; 11 Department of Precision Medicine, Geneus Health LLC, San Antonio, TX, USA

**Keywords:** Neurotransmitter signaling, Reward cascade, Opioid epidemic, Metenkephalin

## Abstract

We are faced with a worldwide opiate/opioid epidemic that is devastating. According to the Centers for Disease Control and Prevention (CDC), at least 127 people, young and old, are dying every day in America due to narcotic overdose. The Food and Drug Administration (FDA) has approved Medication-Assisted Treatments (MATs) for opiate/opioids as well as alcohol and nicotine. The mechanism of action of most MATS favors either blocking of dopaminergic function or a form of Opiate Substitution Therapy (OST). These treatment options are adequate for short-term treatment of the symptoms of addiction and harm reduction but fail long-term to deal with the cause or lead to recovery. There is a need to continue to seek better treatment options. This mini-review is the history of the development of one such treatment; a glutaminergic-dopaminergic optimization complex called KB220. Growing evidence indicates that brain reward circuitry controls drug addiction, in conjunction with “anti-reward systems” as the “anti-reward systems” can be affected by both glutaminergic and dopaminergic transmission. KB220 may likely alter the function of these regions and provide for the possible eventual balancing the brain reward system and the induction of “dopamine homeostasis.” Many of these concepts have been reported elsewhere and have become an integral part of the addiction science literature. However, the concise review may encourage readership to reconsider these facts and stimulate further research focused on the impact that the induction of “dopamine homeostasis” may have on recovery and relapse prevention.

## Introduction

The big question is: ***“what is the best way based on scientific evidence to provide dopamine balance in the brains of people involved in addiction treatment and recovery****”?* Although there is no simple answer, because of the enormous efforts made by our national institutes (NIAAA and NIDA), we are making progress.

## Understanding KB220 as a Novel Candidate to Overcome “Anti-Reward”

Since the major goal of this article is to provide the historical underpinnings of the development of a well-researched nutraceutical known as KB220 and associated variants, it seems obvious to provide information regarding the mechanism of action; bioavailability; half-life, and mode of elimination.

### Composition of and preparation of KB220

KB220 is composed of precursor amino acids the building blocks of neurotransmitters and other ingredients that support neurotransmission. The most recent variant of KB220 includes the following ingredients: Thiamine, 15 mg (1033% of daily value); Vitamin B6, 10 mg (500%); Chromium poly nicotinate 200 mcg (166%); a fixed dose combination of amino acids and herbs called Synaptose. Synaptose contains DL-phenylalanine, L-tyrosine, Passion flower extract; L-glutamine; 5-hydroxytryptophan (5-HTP); Thiamine hydrochloride; Pyroxidal-5-phosphate; Pyridoxine HCl and a composite containing Arabinogalactans, N-acetylglucosamine, Astragalus, Aloe vera, Frankincense resin, White pine bark extract, N-acetyl-cysteine (NAC) and Rhodiola.

### Mechanism of action

**Figure 1:**
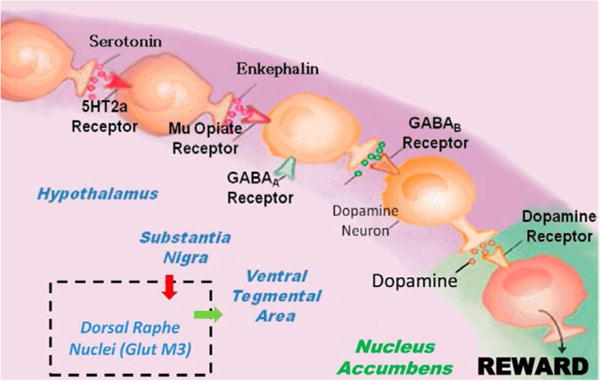
It is an illustration of the Brain Reward Cascade, which involves the release of serotonin at the hypothalamus, where it stimulates enkephalin. The enkephalin then inhibits GABA at the substantia nigra, which, in turn, regulates the amount of dopamine released at the nucleus accumbens (or “reward site”). The dopamine originates in the VTA. Various receptors (including 5HT2a receptors, μ-opiate receptors, GABAA receptors, GABAB receptors, and dopamine receptors) are utilized in the reward cascade. Recent evidence demonstrates the role of the dorsal raphe nuclei in this cascade [85]. It is well known that, under normal conditions, dopamine in the nucleus accumbens through a number of cascading events and neurotransmitter interaction works to maintain a person’s normal drives [86](with permission).

Firstly, regarding the mechanism of action, KB220 ingredients provide some interesting properties which can assist in the induction of “dopamine homeostasis.” To appreciate this, familiarity with the ideas related to reward processing especially the Brain Reward Cascade (BRC) is essential (Figure 1). The precursor 5-HTP provides the needed amino-acid to synthesize serotonin in the hypothalamus. Serotonin synthesis is boosted by the trace metal chromium which acts on insulin–receptors to increase receptor sensitivity, altering the carbohydrate ratio in the periphery to favor the substance tryptophan found in the liver. Tryptophan delivered to the brain is increased by 33% in this way. The addition of L-phenylalanine, a precursor to dopamine, can increase the synthesis of dopamine at the Ventral–Tegmental Area (VTA) by 20%. L-glutamate is converted to GABA at the substania nigra. The use of D-phenylalanine (a known enkephalinase inhibitor) prevents the catabolism of methionine enkephalin and other endorphins by inhibiting the enzyme known as enkephalinase and enhances the activity of natural peptidyl opiates in the hypothalamus. The substance NAC is added to KB220 to help drive the glutaminergic system at the VTA to release dopamine at the Nucleus Accumbens (NAc) the reward site of the brain. Rhodiola was added to the formula because it has been shown to inhibit Catecholamine–Methyl-Transferase (COMT) thereby enhancing the release of dopamine into the synapse to combine with the post dopamine D2 receptor. Rhodiola is also known to inhibit mitochondrial MAO-A which serves to allow more residual dopamine to be transferred to the vesicles of presynaptic neurons (D1–D9) for higher dopamine quanta release. All of these ingredients in KB220 promote a balance across the brain reward circuitry. The various immune boosters like Arabinogalactans, N-acetylglucosamine [1], Astragalus, Aloe vera, Frankincense resin, White pine bark extract may have anti-cytokine qualities which could offset hormones responsible for stress induction and “anti-reward” properties.

## Bio-availability of Amino Acids

Primarily KB220 relies on the benefits of the precursor aminio-acids and it behooves us to present bioavailability and elimination information. Parameters differ for transporting precursor amino-acids like L-glutamate and L-tryptophan across the Blood Brain Barrier (BBB), a low–capacity independent carrier system [2]. Certainly, non-polar compounds like 5–HTP will penetrate the BBB whereas polar compounds like serotonin will not be easily absorbed. Interestingly, stress increases the permeability of the BBB, so pertinent precursor amino acids like tyrosine and L-phenylalanine for the synthesis of brain dopamine would be favored. It is also important to recognize limitations and competitiveness regarding neutral amino acids penetrating the BBB.

**Figure 2:**
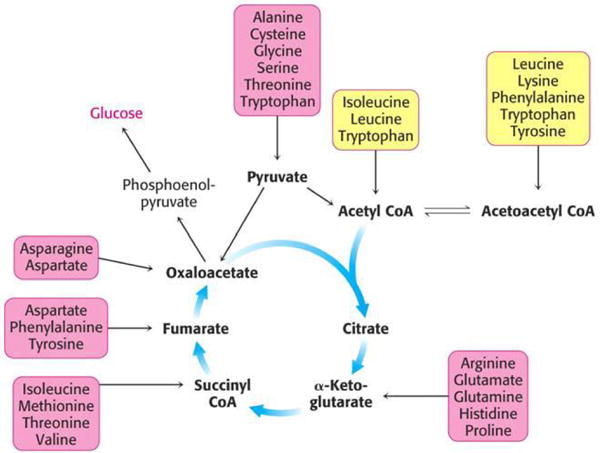
Elimination of amino-acids. The carbon skeletons of amino acids are broken down into metabolites that can either be oxidized into CO_2_ and H_2_O to generate ATP, or can be used for gluconeogenesis. The catabolism of amino acidsaccounts for 10 to 15% of the human body’s energy production. Each of the 20 amino acids has aseparate catabolic pathway, yet all 20 pathways converge into 5 intermediates, all of which can enter thecitric acid cycle. From the citric acid cycle the carbon skeletons can be completely oxidized into CO_2_ or diverted into gluconeogensis or ketogenesis. Glucogenic amino acids are broken down into one of the following metabolites: pyruvate, α-ketoglutarate, succinyl CoA, fumarate or oxaloacetate. Ketogenic amino acids are broken down intoacetoacetate or acetyl-CoA. Larger amino acids, tryptophan, phenylalanine, tyrosine, isoleucine andthreonine are both glucogenic and ketogenic. Only two amino acids are purely ketogenic they are lysine and leucine. If two of the amino acids are purely ketogenic and five amino acids are both ketogenic and glucogenic, than that leaves 13 amino acids that are purely glucogenic: Arginine, Glutamate, Glutamine, Histidine, Proline, Valine, Methionine, Aspartate, Asparagninen, Alaanine, Serine, Cysteine, and Glycine [87].

The half-life of amino-acids is approximately four hours so daily dosage requirements of KB220 is in equal divided doses usually morning and night without food. The mode of elimination of amino-acids is illustrated in Figure 2.

## The Search that Found Evidence for Addiction Treatment

The fifty-year journey began in the late 60’s, and 70’s when one of us (KB) was a post-doctoral fellow at Southwest Foundation for Biomedical Research in San Antonio, Texas. In 1964 Blum revealed that dopamine could control tremors in the periphery of cats [3]. It was one of the few papers to help understand the role of dopamine deficiency in Parkinsonism.

In 1968, funded by a grant from the National Institute on Alcohol Abuse & Alcoholism (NIAAA) for animal research, Blum and Geller looked into the role of neurotransmitters in stress and aberrant alcohol drinking. The focus was on the role of stress-induced changes in brain neurochemistry. The discovery that intense stress-related behavior in rodents was associated with reduced brain serotonin was pivotal in the development of KB220. The Geller and Blum laboratory was the first to look into darkness-induced drinking based on the effect of pineal gland melatonin [4]. Blum and associates continued their research on alcoholism and the pineal gland. During this time, the concept of shared neurochemical mechanisms between alcohol and opiates was developed and presented to the scientific community [5]. This research was the first to show that the narcotic antagonist naloxone could block alcohol-induced sleep time and alcohol dependence in mice [6]. These early findings although controversial, led to the clinical development of Vivitrol (naltrexone), and more recently Suboxone (buprenorphine/naloxone) used currently as an FDA approved pharmaceutical to treat both alcoholism as well as opiate addiction [7]. Similarly, reduce alcohol withdrawal symptoms resulted from the use of both dopamine and morphine [5, 8].

**Figure 3:**
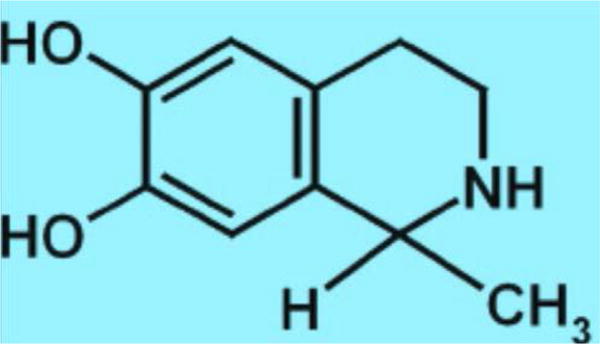
Isoquinolone salsolinol. Chemical structure of Isoquinolone Salsolinol a consequence of condensation of dopamine and acetaldehyde and an agonist at the opiate receptor a brain mechanism shared by alcohol and opiates (internet image).

Others evaluated Isoquinolones, a by-product of the combination of the acid form of alcohol and dopamine, in alcoholism [9, 10]. They found that when a substance formed in the brain when one drinks alcohol, resembles opiates found in poppy plants, Figure 3.

This connection led to the unconventional idea that the “junkie” in the street looking for a heroin fix is neurochemically similar to an alcoholic downing five martinis during lunch. Laboratory work during the next five years was devoted to the commonality between the neurochemical effects of two seemingly different chemicals: alcohol and morphine. During this time, the first evidence to identify an isoquinolone metabolite in brains of mice after ethanol ingestion was observed [11]. This finding led to a notion of shared brain mechanism that occurs in both ***alcohol and opiate*** addiction. The mechanism first described in an edited book published in 1978 [12] was recently expanded on in an article with Gold and others [13]. The importance of this work was that it set the stage for understanding the basis of cross-addictions and poly-drug abuse.

The fundamental discovery of the opiate receptor published in *Science* advanced addiction science [14]. Soon after, endogenous brain peptides, meaning these peptides that occur naturally within the body were discovered eventually called endorphins, a name first coined by Eric Simon [15–17]. Following these very exciting and influential findings, Blum’s group dedicated their research to exploring endorphin function, a critical factor in the actual development of KB220.

**Figure 4:**
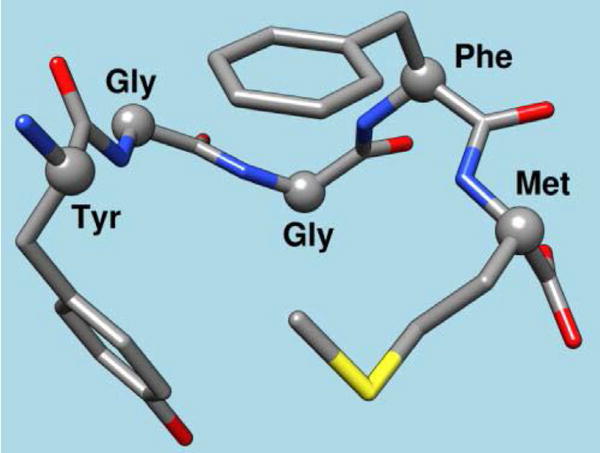
Chemical structure of met-enkephalin. It is an image ofthe natural brain opiate peptide – methionine–enkephalin (METENK) (internet image).

This very first effort demonstrated the unequivocal role of one endorphin-like brain substance called Methionine– Enkephalin (METENK) in alcohol addiction [18, 19]. The results showed that the brain’s content of METENK was in proportion to alcohol intake in mice genetically bred to love or hate alcohol. Specifically, low METENK caused high alcohol drinking, while high METENK induced low alcohol intake. At that time, the thinking was that if genes are indeed one reason why people cannot control their drinking, it could, in part, be due to low levels of morphine peptides. One solution to this problem then would be to find a way to overcome low endorphin levels (Figure 4).

However, before exploring this possibility, the question was asked: “what if the problem was environmental as well as genetic?” In 1982, seminal work published in Science demonstrated that alcohol-loving Golden Syrian hamsters drinking alcohol freely for one year had a very significant reduction of leucine-enkephalin synthesis (production) in the striatum, a brain region involved in craving behavior. One hamster year was the equivalent of 20 years in humans [20]. This finding has since been confirmed in humans and shown by others to occur with chronic intake of opiates, diazepam, and cocaine [21]. Most recently, Howard Field’s group further confirmed these earlier findings [22]. These findings lent support to the idea that all addictions share brain mechanisms (in this case, low enkephalins) [22–25].

In the mid 80’s, Blum’s group was the first to show that injections of enkephalamide (an analog of enkephalin that slows the breakdown of methionine-enkephalin) into the cerebellum induced a significant reduction of alcohol intake in high ethanol seeking C57/blk mice. All of these studies pointed to the idea that low endorphins in the brain lead to high alcohol drinking [26]. So, essentially, one way to prevent this high alcohol intake would be to find a way to increase the brain levels of endorphins.

The idea of ***“pharmacogenomic engineering”*** followed these central findings [26]. A substance D-phenylalanine known to block the biological activity of an enzyme called enkephalinase which brakes down enkephalin by slicing up the five amino acids string (Figure 4) was used to stop the destruction of enkephalin. D-phenylalanine, was a good candidate because, in the 1930’s, it was touted as an inhibitor of the enzyme carboxyl-peptidase, at the time having nothing to do with brain opioids or brain opium. Based on this understanding and the earlier work by others [24], we decided to evaluate D-phenylalanine as a potential anti-alcohol agent.

The administration of D-phenylalanine raised endorphin levels in both the pituitary and the striatum of alcohol-loving c57/blk mice after 18 days. The D-phenylalanine changed mice that were genetically prone to seek alcohol to reduce their alcohol intake so that it was similar to the levels of non-preferring alcohol-hating DBA mice. This work provided the starting point for the role of enkephalinase inhibition as a therapeutic agent for the treatment of alcoholism which was published in the journal Alcohol [26]. Since then little effort has been made by pharmaceutical companies to develop enkephalinase inhibition as a viable anti-alcohol therapeutic agent.

The development of KB220 continued in 1982 with the realization that as well as enkephalinase inhibition, an anti-alcoholic agent must include select precursor neurotransmitter-based amino–acids, and other inhibitors of mitochondrial and synaptic enzyme-catabolizers that destroy brain chemical messengers, such as serotonin and dopamine. For example, D-phenylalanine has a high affinity to the enkephalinase enzyme and instead of binding to methionine–enkephalin preferentially bound to the D-amino acid, at the glycine-phenylalanine binding site (Figure 4).

Following many attempts, the first ever ***“neuronutrient”*** was formulated, initiated a successful reduction of heavy drinking in a male alcoholic. Further reiterations led to the successful prevention of serious drinking in a female alcoholic. For the next eight years, during the first commercialization of KB220, this neuronutrient was used in more than 1,000 treatment centers in the USA [27]. Blum’s group continued to develop related formulations for cocaine dependence, opiate dependence, and even obesity [28–30]. The FDA released information that they later retracted, that identified L-amino-acid tryptophan, as the cause of 35 deaths due to Eosinophilia in some batches. L-tryptophan was an ingredient in the formulation, and KB220Z formulations were withdrawn from the consumer marketplace for a time.

This research continued, and with Gerald Kozlowski of Southwestern Medical School in Dallas, Texas, many scientifically sound research articles were reviewed, and the various ways that neurotransmitters interact within the brain were tracked [31]. With this information, a detailed map that described the reward circuitry of the brain was developed. This basic conceptual framework termed ***“Brain Reward Cascade”*** (BRC) described shared neurochemical correlates between drugs [32] and as common to all addictive behaviors [33, 34]. This idea with recent modifications has stood the test of time and was supported by many scientists across the world over the last 25 years [25]. It is used as a blueprint of neurotransmitter interaction and the subsequent workings of the reward system and rewards dependent behaviors, see Figure 1.

The basic tenant of this work is that the feeling of well-being can be achieved only when the dopamine molecule is released in the nucleus accumbens at balanced “homeostatic” levels. Any deviation causes ***“dopamine resistance”*** and as such, could result in cravings, whether liking or wanting [35, 36]. Also, excessive dopamine can lead to schizophrenia [37] and too little dopamine could lead to unhappiness, anhedonia or depression.

Many research papers related to clinical outcomes, during this period, including double–blind placebo investigations appeared in the scientific literature. The response from the recovery community was positive, and as such, amino-acid therapy for the treatment of drug addiction was born. These developments are detailed in a book, Alcohol and the Addictive Brain [21].

## Genetics and Epigenetics of Addiction

The research effort then turned to investigating the well-known supposition that alcoholism is an inheritable disorder. Studies that specifically related any gene(s) with this familiar global problem were lacking. Blum and associates with Ernest P. Noble, former director of the NIAAA and Psychiatry Professor at UCLA, looked for genes that were involved in the BRC. Analysis of brain tissue from alcoholics (80% cirrhosis of the liver) was compared with tissue from non-alcoholics. Restriction Fragment Length Polymorphism (RFLP) techniques were used to discover the first ever genetic association with alcoholism published in JAMA [38]. Initially, the now famous genetic association of the dopamine D2–receptor gene (DRD2) A1 allele (variant) with severe alcoholism, was met with worldwide controversy [39] Genetic</keyword><keyword>*Polymorphism, Restriction Fragment Length</keyword><keyword>Receptors, Dopamine/*genetics</keyword><keyword>Receptors, Dopamine D2</keyword></keywords><dates><year>1992</year><pub-dates><date>Feb 1</date></pub-dates></dates> <isbn>0006-3223 (Print. Now, this discovery is considered a breakthrough in addiction medicine and has been investigated in 4,300 scientific studies cited in PUBMED (04-1-17). The JAMA study was seminal for the entire field known as “Psychiatric Genetics.” Noble, Blum, and associates did a binding study of the same brain tissue and discovered that compared to carriers of the A2 allele, DRD2 A1 carriers have a 30–40% lower density of D2 receptors [40].

Hypodopaminergic function found in carriers of the A1 allele leaves them at high risk for all addictive behaviors, both substance, and non-substance-related. Indeed, the DRD2 gene is a “reward gene” considered to have a major impact on all reward behaviors and not specific to alcohol, as first stated in our JAMA paper. After the association had been announced, a Gallup poll revealed that the majority of Americans now thought that alcoholism as genetically based rather than a moral failure.

## Common Neurogenetic and Neurobiological Mechanisms and Reward Deficiency Syndrome

In 1995, Blum was the first to conceive the idea of ***“Reward Deficiency Syndrome”*** (RDS), based on a new understanding of the importance of hypodopaminergic traits and states and the nature of neurogenetic and neurobiological mechanisms that are shared across the major abusable drugs. The umbrella term RDS term covers all compulsive and impulsive behaviors, drug and non-drug related, that share the brain chemistry of addiction. As well as addiction, compulsive and impulsive conditions include, attention deficit disorder, substance abuse, food bingeing, pathological gaming, internet addiction, and sex addiction, all having a shared hereditary foundation [41].

The Reward Deficiency Syndrome (RDS) concept was first described in a general article in American Scientist [41] and today, over 599 articles are listed in PUBMED (04-2-17) that deal with “Reward Deficiency,” another 1,113 articles deal with “Dopamine Dysregulation,” and 114 with RDS. In fact, RDS is currently found and defined in Microsoft Word. RDS is included in SAGE Encyclopedia of Abnormal Psychology and Mental Illness 2017. The importance of dissecting the role of dopamine into “wanting” and liking,” has been explored in a body of work [36]. However, these concepts dovetail into the RDS model [35], adopted as part of ASAM’s new definition of addiction in 2011.

Statistical analysis published in the Royal Society of Medicine demonstrated that carriers of the DRD2 A1 allele variant have a predictable 74.4% risk for RDS [42]. Of course, this does not mean an individual is doomed due to predisposing genetics. The environment (through epigenetics) has a 30–50% chance to prevent the expression of these risky gene variations [43]. The influence of the environment on gene expression has been better-understood thanks to the work of Eric J. Nestler and others [44]. The work of Mark Gold sparked many basic and clinical concepts that are incorporated in addiction medicine today [13]. The RDS concept expanded on the ***“dopamine depletion hypothesis”*** for cocaine abuse proposed by Dackis & Gold [45]. Gold’s group suggested the clinical utilization of naloxone in the treatment of addiction, [46]. They provided the mechanism involved in opiate withdrawal and the use of clonidine [47], and proposed the idea that dopamine agonist therapy should be frontline agents in preventing cocaine addiction and relapse [48]. The RDS concept, whereby food and drugs have similar neurochemical mechanisms, was explored by Gold, Hoebel, and Avena from the late 90s up until the present [49, 50]. In fact, although still controversial, their subsequent work and writings support the view that food can be just as addictive as opiates. Parenthetically, Gold’s recent work on second-hand smoke has paved the way for smoke-free zones across the United States [51].

Presently, along with; Dominion Diagnostics, LLC., Members of their Scientific Advisory Board, and in conjunction with Andrew Smolen, and Brett Haberstick from the Institute of Behavioral Genetics at Colorado University Boulder, Blum’s group is developing the first ever Genetic Addiction Risk Score (GARS). In unpublished work, a carefully designed ten-gene polymorphic panel of reward genes was found to significantly predict the ASI-Media Version alcohol and drug severity scores using patients from seven addiction treatment centers throughout the United Sates. This genetic test will benefit addiction treatment by prediction of genetic risk for the development of addiction (necessary for pain clinics) and stratification of genetic risk for those in recovery [52–54]. This genetic test, which combines addiction risk and P450 metabolic predilection (high or low metabolizes), may launch close to the 25^th^ anniversary of the first association of the DRD2 gene with severe alcoholism.

## Treatment Results with KB220

Relevant papers that explored the mechanisms shared between drug and food addiction, reviewed research into substance vaccination [55], gene therapy [56] and pro-dopaminergic regulation therapy for all RDS behaviors [57] have been published. Since the 80’s up until the present time, Blum’s group has published close to 35 peer-reviewed articles showing clinical benefits of KB220 variants [58] especially for craving behaviors [59]. The Generally Recognised As Safe (GRAS) nutrient ingredients of the most recent variant of KB220 are:

- D-phenylalanine inhibits enkephalinase and increase opioid peptides;
- L-phenylalanine is a precursor to dopamine
- L-tryptophane is a precursor to serotonin
- L-tyrosine is the rate limiting substance for the synthesis of dopamine
- L-glutamine is a precursor for GABA
- Chromium increases the synthesis of serotonin and the sensitivity of insulin receptors
- Rhodiola Rosa inhibits the catalyzing enzymes MAO and COMT
- N-acetylcysteine to balance the glutaminergic pathway
- Pyridoxine is an enzyme catalyst

Blum’s group carried out a series of experiments that used neuroimaging tools to explore the effect of KB220Z. Following the intravenous or oral administration of KB220, they used qEEG analysis and LORETA for an ADHD case study [60]. They found significant regulation of the pre-frontal cortices particularly in the cingulate gyrus (a region for drug relapse) in abstinent psychostimulant abusers [61], alcoholics and opiate addicts [62].

**Figure 5:**
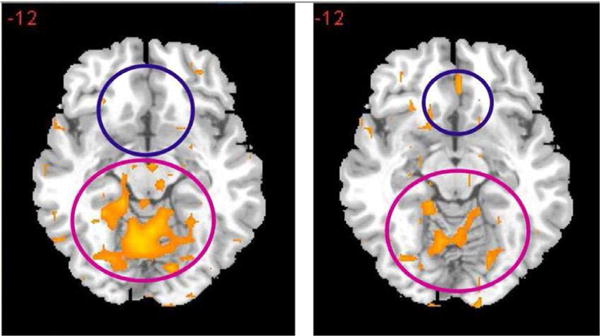
Resting-state fMRI one hour after one dose of KB220 variant. Left side placebo (n=5) Right side KB220 variant (n=5) It represents a fMRI cross-over study in five abstinent heroin addicts receiving either placebo or KB220Z (Synaptose) one –hour prior to testing. It is noteworthy, that following oral KB220Z there is BOLD activation in the NAc and a attenuation of high BOLD activation in the putamen. This illustration suggests a balancing of dopamine function in the brain at the reward site [63] (with permission).

The most prominent finding published to date concerning KB220Z (a glucoside variant) has shown that KB220Z in a placebo-controlled crossover study significantly restored resting state functional connectivity in abstinent heroin addicts. Resting-state functional connectivity was restored across a network that included the posterior cingulate, occipital cortical areas, cerebellum dorsal anterior cingulate, medial frontal gyrus, and nucleus accumbens [63]. Dopaminergic pathways were activated one-hour post-KB220Z, heightened emotionality in the putamen was reduced and resting-sate functional connectivity restored. The findings clearly suggest “dopamine homeostasis” (Figure 5).

It is now accepted that reduced resting-state functional connectivity is a fundamental culprit in addictive behaviors [64]. “Normal” resting state functional connectivity involves “cross-talk” that allows one part of the brain to communicate with another part of the brain, for example, the accumbens the seat of craving talks with the hippocampus where memory resides, and with the Cingulate Gyrus the seat of decision-making. A reduction in this functional connectivity at rest will set-up the individual for addictive-like behaviors.

People, born with normal genetic traits or who have experienced a healthy epigenetic, environmental state, have a brain at rest that is entirely connected, which is a good thing. Alternately, it is now well known that drugs of abuse and other addictive behaviors, like gambling, compulsive sexual behavior, conditions like ADHD, and overeating all reduce resting-state functional connectivity [65, 66]. In other work with non- addicted rats demonstrated that KB220Z compared to a placebo increases in functional connectivity. The activated brain areas included the areas used for memory, decision-making, and craving. These areas include the nucleus accumbens, the cingulate gyrus, anterior thalamic nuclei, hippocampus and also prelimbic and infralimbic loci. Also, evidence for the restoration of resting state functional connectivity was seen as increased neuronal firing, measured by increased connectivity volume [67].

## Treatment Perspectives

Literature supports the idea that people do well in recovery by attending 12 step type programs [both Narcotics Anonymous (NA) and Alcoholics Anonymous (AA)] that focus on both spirituality and fellowship [57]. The evidence for the molecular neurobiology of each step is explained in the Springer Neuroscience Brief book [68].

SAMHSA has been responsible for most of the public funding of drug addiction treatment and has recently changed the language of its grant applications to push the treatment industry away from the previously accepted abstinence model. From the days of (AA) in 1935 to current thinking, some still believe that the abstinence model, which promotes complete restraint from substances including medications prescribed for addiction, is the only acceptable recovery method.

However, a recent initiative has spurred new policies. Within the block grant application for the fiscal years 2016–2017 ($1.8 million was awarded in 2015), SAMHSA now encourages the rejection of the abstinence policy and requires the option of Medication-Assisted Treatment (MAT) in clinical settings. Buprenorphine/naloxone combinations for opioid addicts have been approved by the FDA the short-term treatment with continued restrictions on patients per practitioner. However, the vast majority of rehabilitation facilities across America do not offer such care. While the FDA has approved MAT for alcoholism, opioid dependence, and even nicotine abuse, there is no approved MAT for cocaine and marijuana abuse. Even NIDA and NIAAA understand that MAT alone is not optimal and continue their efforts to find even better treatments.

America is in the middle of a tremendous heroin and prescription opioid epidemic that is targeting youth both white and colored and debilitating future generations. Notably, heroin-related overdose deaths almost quadrupled between 2002 and 2013, according to The Centers for Disease Control and Prevention (CDC). In fact, National Drug Control Policy Director Michael Botticelli stated that the U.S. government would make drug court funding conditional on states’ adherence to scientifically-supported treatment rather than ideologically motivated treatment exemplified by the abstinence model. However, Christie/Trump model targeting borders and reducing insurance coverage for the treatment of SUD could be diasatorious. Instead targeting pharmaceutical prescription abuse (297 million in 2016) seems more parsonomuous.

Indeed, progress in scientifically proven treatments may pave the way for even better long-term less aversive therapies. However, we do know that most FDA approved drugs favor blocking dopamine (are predominantly dopamine antagonists). There is also some evidence that when long-term treatment with buprenorphine/naloxone combinations is compared to short-term treatment, no significant benefit in their resultant clinical outcomes is observed. Studies using fMRI reveal almost 98% saturation of Mu opiate receptors with 16mg of Buprenorphine [69, 70] and long-term use of buprenorphine/naloxone combinations (like Suboxone) can lead to common withdrawal symptoms including suicidal ideation [71].

Over years of research, many including Nora D. Volkow (Director of NIDA), George Koob (Director of NIAAA) and our group have argued that “Dopamine Function” is an important cornerstone for a healthy and happy life [72]. If we accept this idea, then it makes little sense to block or suppress dopamine activity long-term, except to achieve “psychological extinction.” Interestingly, we have demonstrated that in fact, long-term utilization of buprenorphine/naloxone combinations can induce a flattening affect in individuals’ personality characteristics [73].

While there is no magic solution, Mark Gold and associates came close when they proposed the use of Bromocriptine, a robust D2 agonist, to treat cocaine addiction [74]. This new finding did not usher in major change as it was found that chronic use of Bromocriptine reduced numbers of D2 receptors. However, this evidence, along with earlier work from Blum’s laboratory, suggested that rather than, direct “dopamine agonist therapy” indirect pro –dopamine regulation, should be embraced in the long-term treatment of addiction. Individuals exhibiting RDS behaviors have been shown to possess low dopaminergic function, due to either stress, the toxic effect of substances (epigenetics) or genetics. There are many addiction risk gene variants called polymorphisms. They are variations of the reward genes, like, for example, DRD1-4; DAT1; Serotonin transporter, GABA, Mu-Opiate receptor, COMT, and MAO that are responsible for hypodopaminergic gene functions and victims of RDS may have varying numbers of these polymorphisms [62].

So while it is important to consider short-term dopamine antagonistic therapy as espoused by FDA approval of MAT drugs, long-term use cannot be recommended. Understandably, while many of the proponents of current MAT would argue against this premise, we are looking for ways to normalize dopamine regulation. Imagine a solution that could provide regulation or “normalization” of dopaminergic function leading to what has been called “dopamine homeostasis?” In this way, balancing the brain’s neurotransmitter signaling should work best regardless of whether the trait or state is hyperdopaminergic or hypodopaminergic [75].

In fact, only a small percentage of treatment centers currently embrace this concept by offering dopamine-boosting modalities. Practices like meditation and yoga exercise, sound and music therapy, brain spotting and behavioral, cognitive therapy, trauma therapy, and adhering to a dopamine-friendly diet are dopamine boosting. Holistic approaches require investigation for direct evidence for dopamine enhancement effects, however, the literature shows a 65% increase in neuronal dopamine with practices like Yoga and Mediation [76] and certain healthy low glycemic foodstuffs, like fish oils, are known to boost dopamine function.

The first statistical analysis showing both compliance to FDA-approved MAT and abstinence from drugs of abuse during treatment used data from Comprehensive Analysis of Reported Drugs (CARD) provided by Dominion Diagnostics. The study involved urine drug screens from thousands of patients in Chemical Dependency Programs from six states was published in PLoS One [77]. The conclusion was simple: both compliance and abstinence in these patients was improved. Despite MAT’s short term efficacy; enhancing dopamine function (not dopamine blockade) in the long term may better serve those in recovery.

**Figure 6:**
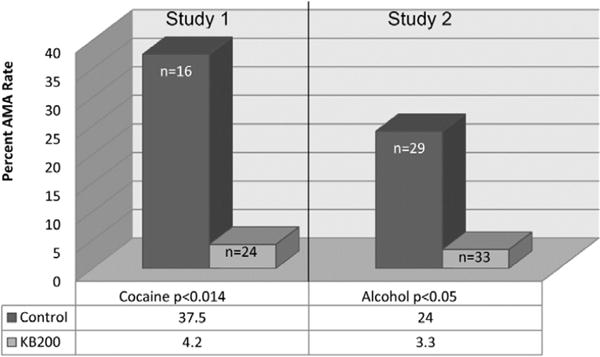
Discharge Against Medical Advise (AMA) control vs. KB220 variant. Study 1 (p<0.014) [79] Study 2 (p<0.05) [28]

**Figure 7:**
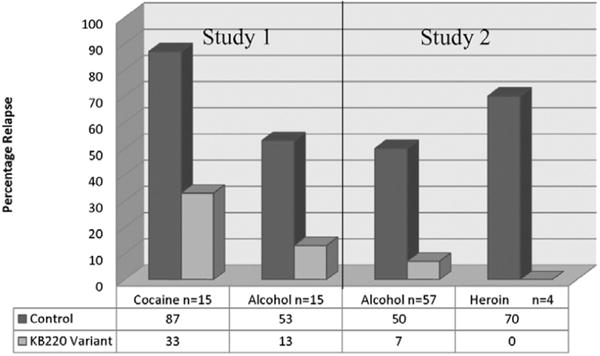
Relapse control vs. KB220 variant. Study 1 Brown et al. Journal of Psychoactive Drugs 22: 173–187 (1990) after 10 months (p<0.001) [29]. Study 2 Chen et al. Advances in Therapy 24: 402–414 (2007) after 12 months (p<0.001) [59].

Regarding relapse vulnerability, there is evidence of an increased risk for relapse to carriers of the A1 form of the of the DRD2 gene have [78] and if they enter treatment, they have a higher chance that they will leave against medical advice (AMA). However, research has shown a significant reduction in not only AMA rates [28, 79] (Figure 6) but reduce relapse (Figure 7) [29, 59] with the use of KB220Z.

Along these lines glutaminergic–dopaminergic optimization complex therapy (KB220), has been well-researched; in many clinical trials. KB220Z has been shown to provide gentle activation of dopamine across the brain reward circuitry in abstinent heroin and abstinent psychostimulant addicts [63]. Significant resting state functional connectivity increases have also been demonstrated in animal models using state of the art resting state fMRI measurements [64]. Continued research on this topic may result in evidence that long-term dopamine agonist therapy with a KB220 variant leads to necessary “dopamine homeostasis,” which may be the missing link to all RDS addictive behaviors both substance and non-substance-related [80].

This summary of the research results provides substantial scientific support for the use of KB220 formulations based on almost 40 years of research. Indeed, we have recently demonstrated increased resting state functional connectivity and induction of physiological changes in the brain (neuroplasticity) [67].

Moreover, along with millions of recovering addicts here in the United States, low dopamine function may be prevalent in the general non-addicted population in part because of either their genetics or environmental pressure (stress). Notably, low dopamine function has been definitively linked in studies to reduced cognition (164) decision making (45) and exercise activity (73); excessive cravings (241); reduced performance (291) and loss of memory (199) aging (218); vulnerability to stress (374); overeating (162) sadness (257); poor relationships (36); and lack of well-being (984).

The proposed “Reward Deficiency System Solution” includes:

1. GARS;
2. Drug urine monitoring for both Compliance to MAT and Abstinence from psychoactive drugs;
3. Long-term use of glutaminergic-dopaminergic optimization complex therapy to induce dopamine homeostasis;
4. Polymorphic DNA-directed mRNA genetic expression profiling.

One frequent question concerning the use of KB220 in the treatment and prevention of relapse for all RDS behaviors is; “Will KB220 be required to be taken life-long- to maintain the balance in the brain reward system?” The answer has two parts. Genetically induced RDS like genetically induced diabetes which is a life-long condition will require lifelong treatment. However, although RDS can be caused by inheritable factors like having the A1 allele of the DRD2 gene amongst other reward gene polymorphisms, it can also be caused by epigenetic effects like stress (anti-reward) as well as the toxic effects of substances. Epigenetic effects can last up two generations [82], although, milder epigenetic effects may not require life-long KB220 neuronutrient support. Unlike powerful pharmaceuticals such as D2 receptor agonists, like Bromocriptine, with side effects including chronic down – regulation of DRD2 receptors, KB220 has been shown to be side effect free (except for an occasional headache) with no associated tolerance or withdrawal reactions [81].

## Conclusion

The hope is that leading minds in addiction medicine, clinics, and interested scientists will come to recognize that to boost (or modulate), rather than blocking dopamine function will eventually lead to better quality of life for all. Relapse prevention and long-term treatment with dopamine regulation may provide a more comprehensive and humane way of treating addiction disorders. The scientific and medical community is challenged here to admit that our current treatment protocols are severely ineffective (locking people into addiction rather than treating the root cause). It is clear that addiction treatment requires a more comprehensive solution. These challenges in no way negate the enormous efforts of countless people who have unselfishly given so much to the field. While the FDA’s push for MAT and the use of off-label Gabapentin, Topiramate, Ketamine and other drugs to alter the brain reward circuitry is understood, the potential for reducing further, needed dopamine with these pharmaceuticals in the chronic treatment of RDS behaviors, is indeed counter-intuitive. The message is to activate (balance), not block, dopamine function in the reward circuitry of the brain in the long-term [82]. We are on the correct path, and we must carry out needed research on the potential of gentle induction of “dopamine homeostasis” by balancing serotonergic, endorphinergic, cannabinergic, glutaminergic, and dopaminergic mechanisms [83, 84].

Lastly, in addressing the concern about “An American Opioid Epidemic” the recent restrictions on prescription opioid use in chronic pain conditions by the Center for Disease Control (CDC, 2016) are appropriate**.** In fact, the Obama selection of the former US Surgeon General has encouraged physicians across the United States to curtail the prescription of powerful narcotic pharmaceuticals and find new ways to screen for a predisposition to high addiction risk before treating pain with these narcotic agents. The conception of screening for addiction risk using a panel of reward genes such as the GARS would be one intervention that might be useful in averting some of the pain involved in the prevention and treatment of this opioid epidemic [80] leading to precision medicine.
